# Postoperative complications after vacuum-assisted excision of benign breast tumors and their associated risk factors

**DOI:** 10.1097/MD.0000000000047791

**Published:** 2026-03-13

**Authors:** Yanfang Tan, Qiong Xu, Hao Wu

**Affiliations:** a Department of Thyroid and Breast Surgery, Pingxiang People’s Hospital, Pingxiang City, China; b Ganxi Hospital, Pingxiang City, China.

**Keywords:** benign breast tumor, complications, nomogram, predictive model, risk factors, vacuum-assisted excision

## Abstract

This study aims to investigate the incidence of postoperative complications following vacuum-assisted excision (VAE) of benign breast tumors and identify the associated risk factors, in order to support clinical risk assessment and perioperative management. A retrospective review was conducted of 100 female patients who underwent ultrasound-guided VAE for benign breast lesions at our institution between December 2023 and December 2024. Patients were categorized into a complication group (n = 30) and a non-complication group (n = 70) based on the occurrence of complications within 30 days postoperatively. Clinical and procedural characteristics were compared between the 2 groups. Univariable and multivariable logistic regression analyses were performed to identify independent risk factors. A predictive model was established and evaluated using the receiver operating characteristic curve and calibration curve. A nomogram was constructed for individualized risk prediction. The overall 30-day complication rate was 30.00%. The most common complications were hematoma (12.00%), subcutaneous ecchymosis (11.00%), pain (9.00%), skin depression (5.00%), and wound infection (1.00%). Univariable analysis indicated that tumor size, number of lesions, distance from the skin, surgeon experience, and compression time were significantly associated with postoperative complications (*P* < .05). Multivariable analysis identified tumor size (odds ratio [OR] = 2.12, 95% CI: 1.12–4.00), distance from the skin <5 mm (OR = 2.62, 95% CI: 1.08–6.35), compression time <10 minutes (OR = 2.80, 95% CI: 1.18–6.63), and surgeon experience <3 years (OR = 2.42, 95% CI: 1.02–5.73) as independent risk factors. The model demonstrated good discriminative ability (area under the curve = 0.826, 95% CI: 0.732–0.921) and acceptable calibration (Hosmer–Lemeshow *P* = .239). The nomogram provided an intuitive visualization for individualized complication-risk prediction. Complications after VAE for benign breast tumors are relatively common, with hematoma, ecchymosis, and pain being the most frequent. Larger tumors, superficial lesions, insufficient postoperative compression, and limited surgeon experience significantly increase the risk of complications. The prediction model and nomogram offer a practical tool for individualized perioperative risk assessment and complication prevention.

## 1. Introduction

Benign breast tumors—such as fibroadenomas and intraductal papillomas—are highly prevalent among women, and many patients seek minimally invasive removal due to progressive enlargement of the mass, pain, or cosmetic concerns.^[1,2]^ With advancements in ultrasound and stereotactic guidance, vacuum-assisted excision/breast biopsy (VAE/VABB) has become a widely accepted approach for the management of benign breast lesions. It offers advantages including minimal invasiveness, short recovery time, and favorable cosmetic outcomes, and is supported by multiple systematic reviews and clinical consensus statements. Meta-analyses involving large cohorts have demonstrated consistent rates of complete excision and overall procedural safety for VAE/VABB in benign breast disease.^[3]^

Despite its generally favorable safety profile, VAE/VABB can result in postoperative complications such as hematoma, subcutaneous ecchymosis, pain, and, less frequently, infection or vascular injury.^[4]^ Systematic reviews report pooled incidences of hematoma, pain, and ecchymosis of approximately 9.2%, 8.2%, and 7.5%, respectively, though these estimates vary across studies, likely due to differences in patient selection, needle gauge, and operative technique.^[3,4]^ Although major bleeding complications after ultrasound-guided breast procedures are uncommon, minor bleeding-related events remain the most prevalent and usually resolve without the need for invasive intervention.^[5,6]^

Several clinical and procedural factors have been proposed as potential predictors of complications, including lesion size, needle gauge (with larger needles associated with increased bleeding risk), excised volume, distance from the skin, compression duration, and surgeon experience.^[7]^ However, evidence across studies is not entirely consistent. For example, stereotactic or ultrasound-guided procedures using larger needles (7–9G) may carry elevated risks of hematoma, while some retrospective cohorts have failed to confirm these associations, indicating the need for further validation in diverse populations.^[8,9]^ Larger reviews of image-guided breast procedures also highlight that severe hemorrhagic complications occur in <1% of cases, yet standardized tools for identifying high-risk individuals preoperatively remain lacking.

Recently, attempts have been made to develop prediction models and machine-learning algorithms incorporating clinical and imaging variables to improve risk stratification for common VAE-related complications such as ecchymosis and hematoma. However, these models remain at an exploratory stage, often lack external validation, and are rarely constructed using data from Asian populations.^[10–12]^ Therefore, conducting studies within real-world Eastern clinical settings to systematically characterize the profile of VABB-related complications, identify independent risk factors using standardized definitions, and build clinically applicable visual prediction tools such as nomograms is of substantial clinical importance. Such tools may support preoperative counseling, individualized perioperative planning, and targeted prevention strategies.

In this context, we retrospectively analyzed 100 consecutive patients who underwent ultrasound-guided VABB at our center over the past year. We aimed to describe the 30-day postoperative complication spectrum, identify independent risk factors, establish and internally validate a predictive model, and develop a nomogram for individualized risk estimation, with the goal of providing a practical tool for clinical risk stratification.

## 2. Methods

### 2.1. Study design and patient selection

This study was approved by the Ethics Committee of Pingxiang People’s Hospital. Due to the retrospective and non-interventional nature of the study, the requirement for informed consent was waived. All data were anonymized prior to analysis to protect patient confidentiality. This single-center retrospective cohort study included 100 female patients who underwent ultrasound-guided VAE and were pathologically confirmed to have benign breast tumors between December 2023 and December 2024. All eligible cases were consecutively identified through the hospital’s electronic medical record system.

The inclusion criteria were: preoperative imaging suggesting benign or probably benign lesions (breast imaging-reporting and data system category 3–4a); postoperative histopathology confirming a benign tumor; and a minimum follow-up duration of 30 days with complete clinical data. Patients were excluded if they had postoperative pathology indicating malignant or borderline lesions, a known coagulation disorder or long-term use of anticoagulants, a history of other breast procedures within 30 days before or after VAE, or missing key clinical information.

Because this was a retrospective, non-interventional analysis, all data were anonymized before analysis, and no identifiable personal information was involved; therefore, institutional ethical approval was not required.

### 2.2. Surgical procedure

All procedures were performed by breast interventional radiologists who were certified and trained in standardized VAE techniques. Ultrasound-guided excision was conducted using the West China Zhenxuan vacuum-assisted excision system with a 7-gauge needle. Patients were positioned supine or in a slight oblique position. Following routine skin disinfection and local anesthesia, the needle was inserted from the inferior aspect of the lesion under real-time ultrasound monitoring. Sequential vacuum-assisted cuts were made until complete removal of the lesion was confirmed.

Upon completion, firm manual compression with sterile gauze was applied for approximately 10 minutes, followed by elastic bandage wrapping to maintain adequate compression. Postoperative analgesia, including acetaminophen or nonsteroidal anti-inflammatory drugs, was administered as needed. Patients were instructed to avoid strenuous upper-limb activities and direct breast pressure for 48 hours after the procedure. Details including needle type, excision time, excised volume, and postoperative compression duration were documented in the operative report for all cases.

### 2.3. Outcomes and definitions

The primary outcome was the occurrence of any postoperative complication within 30 days. Complications included hematoma, subcutaneous ecchymosis, pain, skin depression, and puncture-site infection. Hematoma was defined as a palpable fluctuant mass or an ultrasound-detected fluid collection ≥ 2 cm within 48 hours postoperatively. Subcutaneous ecchymosis was defined as bruising appearing within 72 hours and measuring >5 cm in diameter or involving 2 or more quadrants. Pain was assessed using the numeric rating scale (NRS), with NRS ≥ 4 requiring escalation of analgesia classified as significant pain. Skin depression was evaluated by physical examination and photographic comparison during follow-up visits. Puncture-site infection was defined as erythema, warmth, tenderness, or discharge requiring antibiotic therapy or incision and drainage.

Secondary outcomes included the incidence of each complication subtype, management strategies, symptom resolution time, and cosmetic satisfaction.

Potential risk factors were also recorded, including patient demographics (age, body mass index, diabetes, hypertension, and use of antiplatelet or anticoagulant medications), tumor characteristics (maximum diameter, number of lesions, distance from the skin, and lesion location), and procedural factors (needle gauge, surgeon experience, and compression duration). Tumor size was defined as the longest diameter on ultrasound. The distance from the skin was measured as the shortest perpendicular distance on ultrasound and dichotomized at 5 mm. Surgeon experience was categorized as <3 years or ≥3 years of independent practice. Compression time was dichotomized at 10 minutes. Follow-up data were collected through outpatient visits or telephone interviews at postoperative days 1, 7, and 30 to ensure complete complication documentation.

### 2.4. Data management and quality control

Data extraction was performed independently by 2 investigators using a standardized electronic case-report form. Any discrepancies were resolved through verification by a third investigator who reviewed the original medical and nursing records. Continuous variables were tested for normality. Variables with <10% missing data were analyzed using complete-case analysis, whereas those with ≥10% missingness underwent multiple imputation (m = 10) for sensitivity analysis. All data were anonymized and stored in encrypted databases before statistical processing. This study is a retrospective one. All the information of the patients we collected is complete. There is no loss of data or information in this study.

### 2.5. Statistical analysis

All statistical analyses were conducted using SPSS version 26.0 (IBM [International Business Machines Corporation], Armonk) and R software version 4.3.1 (The R Foundation for Statistical Computing, Vienna, Austria). Continuous variables were expressed as mean ± standard deviation and compared using independent-sample *t* tests. Categorical variables were summarized as counts and percentages and compared using the chi-square test or Fisher exact test, as appropriate. Variables with *P* < .05 in univariable analysis were included in a multivariable logistic regression model to identify independent predictors, with odds ratios (ORs) and 95% confidence intervals (CIs) calculated.

A predictive model was developed based on the final regression equation. Model discrimination was assessed using receiver operating characteristic curves and the area under the receiver operating characteristic curve (AUC). Model calibration was evaluated using the Hosmer–Lemeshow goodness-of-fit test and calibration plots. A nomogram was constructed from the regression coefficients to enable individualized prediction of postoperative complications, and a forest plot was generated to visualize the effect sizes of independent predictors. All tests were 2-tailed, with *P* < .05 considered statistically significant.

## 3. Results

### 3.1. Baseline characteristics

A total of 100 female patients who underwent ultrasound-guided VABB for benign breast tumors were included, of whom 70 experienced no postoperative complications and 30 developed at least 1 complication. The mean age of the cohort was 38.64 ± 8.52 years, and the mean body mass index was 23.92 ± 3.21 kg/m^2^. There were no significant differences in age or body mass index between the 2 groups (*P* > .05).

Several clinical and procedural characteristics were significantly associated with postoperative complications. Patients in the complication group had larger tumors (2.27 ± 0.87 cm vs 1.82 ± 0.76 cm, *t* = 2.536, *P* = .013) and a higher proportion of multiple lesions (30.00% vs 12.86%, *χ*^2^ = 3.946, *P* = .047). A greater proportion of these patients had lesions located <5 mm from the skin (36.67% vs 15.71%, *χ*^2^ = 4.951, *P* = .026), surgeon experience <3 years (40.00% vs 18.57%, *χ*^2^ = 4.955, *P* = .026), and postoperative compression <10 minutes (43.33% vs 20.00%, *χ*^2^ = 5.820, *P* = .016) (Table 1).

**Table 1:** Baseline characteristics of patients.

Variable	Without complications (n = 70)	With complications (n = 30)	*t*/*χ*^2^	*P* value
Age	37.59 ± 8.40	41.03 ± 8.38	1.808	.074
BMI (kg/m^2^)	23.47 ± 3.01	24.89 ± 3.37	1.987	.050
Diabetes	4 (5.71)	4 (13.33)	1.573	.210
Hypertension	9 (12.86)	5 (16.67)	0.230	.631
Antiplatelet/anticoagulant use	5 (7.14)	6 (20.00)	3.014	.083
Tumor size (cm)	1.82 ± 0.76	2.27 ± 0.87	2.536	.013
≥2 lesions	9 (12.86)	9 (30.00)	3.946	.047
Distance from skin <5 mm	11 (15.71)	11 (36.67)	4.951	.026
Needle gauge ≥10G	19 (27.14)	14 (46.67)	3.482	.062
Surgeon experience <3 yr	13 (18.57)	12 (40.00)	4.955	.026
Compression time <10 min	14 (20.00)	13 (43.33)	5.820	.016

BMI = body mass index.

### 3.2. Incidence of postoperative complications

The overall 30-day postoperative complication rate was 30.00% (30/100). The most frequently observed complications were hematoma (12.00%) and subcutaneous ecchymosis (11.00%), followed by significant pain (NRS ≥ 4, 9.00%) and skin depression (5.00%). Only 1 case of puncture-site infection occurred (1.00%). Most hematomas and ecchymoses resolved with conservative management, including compression and cold application; only a single hematoma required needle aspiration (Table 2).

**Table 2:** Postoperative complications after VABB.

Complication	Cases (n)	Incidence (%)	95% CI
Any complication	30	30	21.30–39.70
Hematoma	12	12	6.40–19.80
Subcutaneous ecchymosis	11	11	5.60–18.60
Pain (NRS ≥ 4)	9	9	4.20–16.40
Skin depression	5	5	1.60–11.20
Wound infection	1	1	0.02–5.40

NRS = numeric rating scale, VABB = vacuum-assisted breast biopsy.

### 3.3. Univariable analysis

Univariable analysis showed that tumor size (*t* = 2.536, *P* = .013), distance from the skin <5 mm (*χ*^2^ = 4.951, *P* = .026), compression time <10 minutes (*χ*^2^ = 5.820, *P* = .016), and surgeon experience <3 years (*χ*^2^ = 4.955, *P* = .026) were significantly associated with the occurrence of postoperative complications. Multiple lesions (≥2) were also associated with increased complication risk (*χ*^2^ = 3.946, *P* = .047) (Table 3).

**Table 3:** Univariate analysis of factors associated with complications.

Variable	With complications (n = 30)	Without (n = 70)	*χ*^2^/*t*	*P* value
Tumor size (cm)	2.27 ± 0.87	1.82 ± 0.76	2.536	.013
Distance from skin <5 mm	11 (36.67)	11 (15.71)	4.951	.026
Compression time <10 min	13 (43.33)	14 (20.00)	5.820	.016
Surgeon experience <3 yr	12 (40.00)	13 (18.57)	4.955	.026
≥2 lesions	9 (30.00)	9 (12.86)	3.946	.047
Antiplatelet/anticoagulant use	6 (20.00)	5 (7.14)	3.014	.083

### 3.4. Multivariable logistic regression

Variables with *P* < .05 in univariable analyses were entered into the multivariable logistic regression model. Tumor size (OR = 2.12, 95% CI: 1.12–4.00, *P* = .021), distance from the skin <5 mm (OR = 2.62, 95% CI: 1.08–6.35, *P* = .033), compression time <10 minutes (OR = 2.80, 95% CI: 1.18–6.63, *P* = .019), and surgeon experience <3 years (OR = 2.42, 95% CI: 1.02–5.73, *P* = .045) remained independent predictors of postoperative complications. Model fit was acceptable (Hosmer–Lemeshow *χ*^2^ = 4.217, *P* = .239), with a pseudo-*R*^2^ of 0.322 (Table 4).

**Table 4:** Multivariate logistic regression analysis of independent risk factors for postoperative complications.

Variable	β	OR	95% CI	*P* value
Tumor size (cm)	0.752	2.12	1.12–4.00	.021
Distance from skin <5 mm	0.963	2.62	1.08–6.35	.033
Compression time <10 min	1.028	2.80	1.18–6.63	.019
Surgeon experience <3 yr	0.884	2.42	1.02–5.73	.045

### 3.5. Model discrimination and calibration

**Figure 1.**
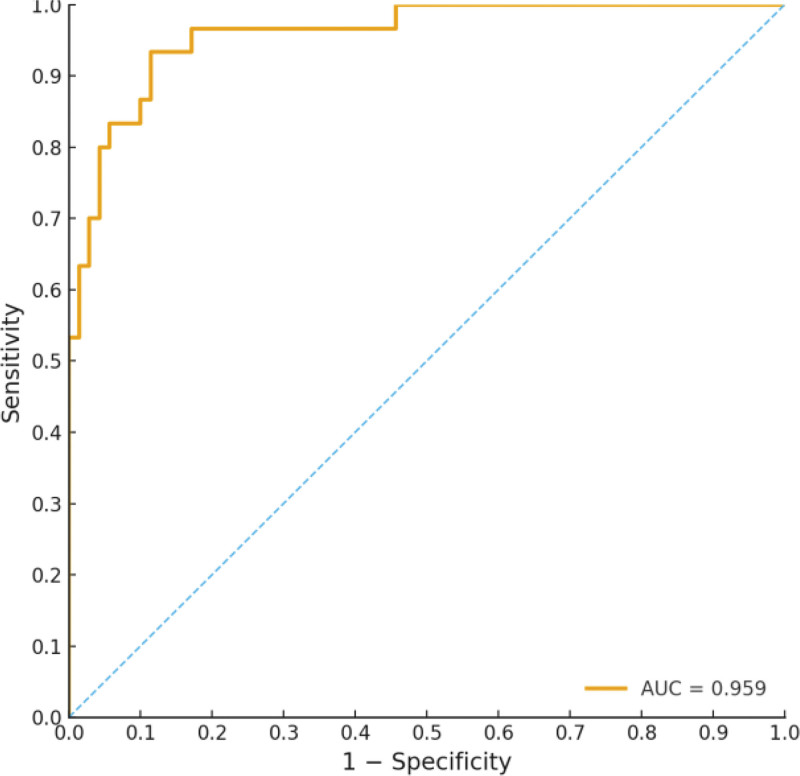
ROC curve for the predictive model. ROC = receiver operating characteristic.

**Figure 2.**
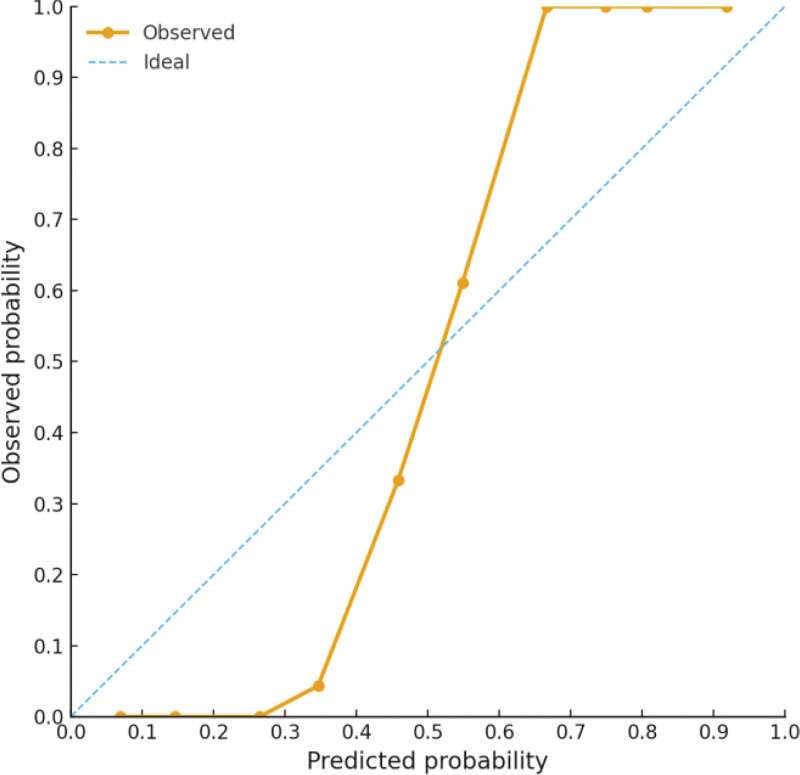
Calibration curve for the predictive model.

The predictive model demonstrated good discrimination, with an AUC of 0.826 (95% CI: 0.732–0.921). At the optimal cutoff, the model achieved a sensitivity of 76.7% and a specificity of 78.6% (Youden index = 0.553) (Fig. 1). Calibration analysis showed strong agreement between predicted probabilities and observed outcomes (Hosmer–Lemeshow *P* = .239), indicating good model calibration (Fig. 2).

### 3.6. Nomogram development and visualization

**Figure 3.**
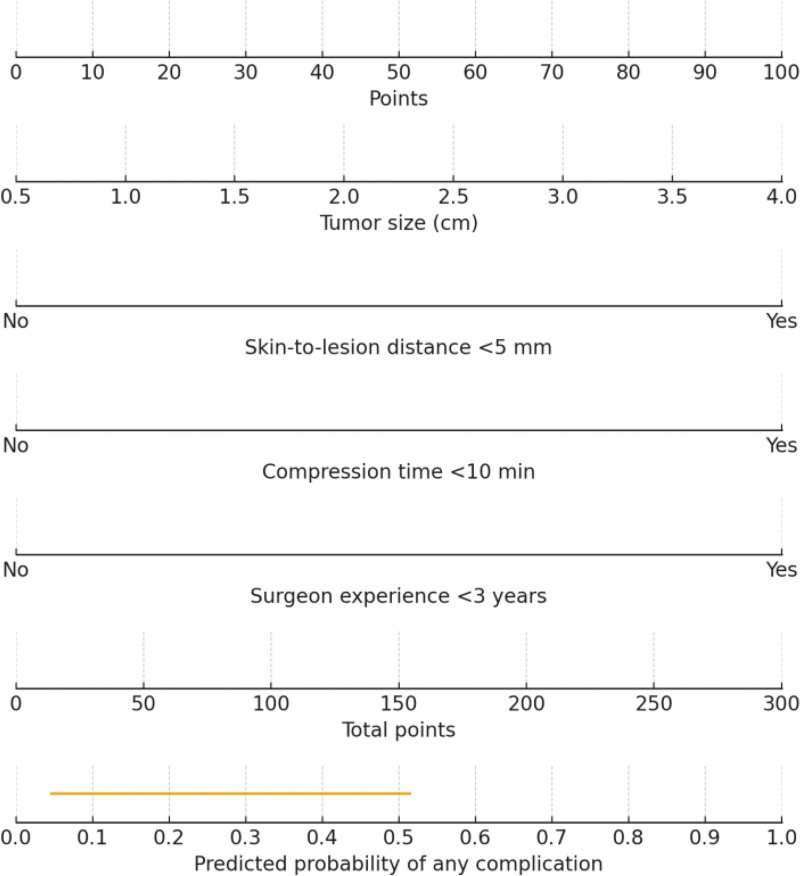
Matplotlib chart.

**Figure 4.**
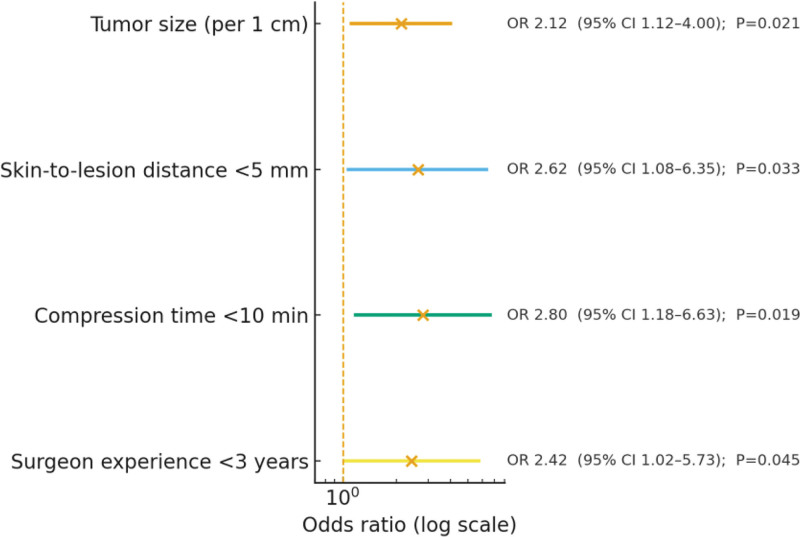
Forest plot of independent risk factors.

To facilitate clinical application, a nomogram was constructed based on the 4 independent predictors (Fig. 3). In the nomogram, tumor size contributed approximately 73 points per 1-cm increase, distance from the skin <5 mm contributed 93 points, compression time <10 minutes contributed 100 points, and surgeon experience <3 years contributed 86 points. Higher total scores corresponded to increased complication risk. For example, a patient with a 2.5-cm lesion located <5 mm from the skin, with compression time <10 minutes and a surgeon with <3 years of experience, would have a total score of approximately 460 points, corresponding to a predicted complication risk >80%. A forest plot illustrating the effect sizes and confidence intervals of the independent predictors is shown in Figure 4.

## 4. Discussion

In this retrospective study of 100 female patients who underwent ultrasound-guided VABB for benign breast tumors, the overall 30-day complication rate was 30.0%. Hematoma (12.0%), subcutaneous ecchymosis (11.0%), and significant pain (9.0%) were the most frequently observed events, whereas skin depression and puncture-site infection occurred less often. Multivariable analysis identified tumor size, distance from the skin <5 mm, compression time <10 minutes, and surgeon experience <3 years as independent predictors of postoperative complications. The predictive model demonstrated good discrimination (AUC = 0.826) and satisfactory calibration (Hosmer–Lemeshow *P* = .239), supporting its potential clinical utility for individualized risk assessment.

The findings of this study are consistent with previous reports. In a large systematic review of VABB for benign breast lesions, Liu et al^[13]^ noted that hematoma and ecchymosis were the most common postoperative complications, with reported rates ranging from 7% to 15%. Chae et al^[14]^ observed that lesion size, needle gauge, and procedure duration were major determinants of hematoma formation, as larger or multiple lesions generally require more excision cycles and result in greater tissue disruption. A prospective study by Yang et al^[15]^ also confirmed that lesions located <5 mm from the skin exhibited a substantially higher hematoma risk, aligning with the present findings. Interestingly, Park et al^[16]^ reported that needle gauge (9G vs 10G) did not significantly influence overall complication rates, whereas sufficient postoperative compression was a key determinant of outcome. Collectively, these studies highlight that, beyond anatomical and lesion-related factors, operator technique and postoperative management play critical roles in minimizing complications.

From a pathophysiological perspective, VABB is a vacuum-assisted rotary cutting technique that may disrupt small vessels adjacent to the excision cavity. Larger lesions require a wider resection cavity, which increases the potential for intraluminal bleeding, particularly when postoperative compression is inadequate.^[17]^ When a lesion lies close to the skin surface, the thin soft-tissue layer between the needle path and the dermis provides limited buffering, making ecchymosis or mild skin indentation more likely.^[18]^ Surgeon experience influences needle-path planning, excision trajectory, and hemostasis proficiency; less experienced operators may have reduced control over intraoperative bleeding. Previous studies have emphasized that standardized compression lasting ≥10–15 minutes, supplemented by elastic bandaging, can substantially reduce the risk of hematoma and ecchymosis.^[19,20]^ These findings underscore the importance of optimal preoperative imaging assessment, appropriate entry-site selection, and adequate postoperative compression to prevent complications in VABB.

The prediction model developed in this study incorporated 4 independently significant risk factors and achieved an AUC of 0.826, comparable to the machine-learning-based model reported by Wen et al^[21]^ (AUC = 0.831). Although machine-learning methods may provide marginal performance gains, nomograms offer advantages in interpretability and bedside applicability, making them valuable tools for preoperative counseling and individualized perioperative planning.^[22]^ To our knowledge, this study is among the first to construct a VABB complication-risk nomogram using real-world data from an Eastern population, contributing novel evidence to the field.

Several limitations should be noted. First, the study was retrospectively conducted at a single center with a relatively small sample size, which may introduce selection bias. Second, some potential influencing factors—such as excised volume, procedural duration, and breast density—were not included in the model and may affect predictive accuracy. Third, only internal validation was performed; external validation in independent cohorts is required to assess generalizability. Finally, mild symptoms such as minor pain or ecchymosis were based partly on medical documentation and patient self-report, which may introduce subjectivity. Future multicenter prospective studies incorporating imaging parameters and patient-reported outcomes are warranted to refine prediction models and further improve the safety and satisfaction associated with VABB procedures. Because this was a retrospective study, information bias and selection bias cannot be completely excluded. Mild complications such as minor pain or limited ecchymosis may not have been consistently documented in medical records or may rely on patient self-report during follow-up, potentially leading to underestimation of their true incidence. Several potentially important variables, including excised tissue volume, procedural duration, intraoperative bleeding, breast density, and detailed antiplatelet or anticoagulant medication history, were not included in the predictive model. Although patients with known coagulation disorders or long-term anticoagulant therapy were excluded, routine use of low-dose antiplatelet agents such as aspirin could not be reliably extracted from retrospective records. The omission of these variables may affect the predictive accuracy and generalizability of the model and should be addressed in future prospective studies.

## 5. Conclusion

VABB is a generally safe and effective minimally invasive technique for the treatment of benign breast tumors; however, postoperative complications remain relatively common. Larger tumor size, superficial lesion location, insufficient postoperative compression, and limited surgeon experience were identified as major risk factors. The nomogram developed in this study demonstrated good predictive performance and may assist clinicians in preoperative risk stratification and individualized perioperative decision-making. Further validation in larger, multicenter cohorts is recommended to enhance the model’s applicability.

## Author contributions

**Conceptualization:** Yanfang Tan, Qiong Xu, Hao Wu.

**Data curation:** Yanfang Tan, Qiong Xu, Hao Wu.

**Formal analysis:** Yanfang Tan, Qiong Xu, Hao Wu.

**Funding acquisition:** Yanfang Tan, Hao Wu.

**Investigation:** Yanfang Tan, Hao Wu.

**Writing – original draft:** Yanfang Tan, Hao Wu.

**Writing – review & editing:** Yanfang Tan, Hao Wu.
